# Acoustic Neurofeedback Increases Beta ERD During Mental Rotation Task

**DOI:** 10.1007/s10484-018-9426-0

**Published:** 2018-12-18

**Authors:** Wioletta Karina Ozga, Dariusz Zapała, Piotr Wierzgała, Paweł Augustynowicz, Robert Porzak, Grzegorz Marcin Wójcik

**Affiliations:** 1 Department of Experimental Psychology, Institute of Psychology, The John Paul II Catholic University of Lublin, Al. Racławickie 14, 20-950 Lublin, Poland; 2 Department of Neuroinformatics, Institute of Computer Science, Maria Curie-Sklodowska University, Akademicka 9/509, 20-033 Lublin, Poland; 3 Department of Psychology, Faculty of Human Sciences, University of Economics and Innovation in Lublin, Projektowa 4, 20-209 Lublin, Poland

**Keywords:** Mental rotation, Beta ERD, Neurofeedback, Engagement index

## Abstract

The purpose of the present study was to identify the effect of acoustic neurofeedback on brain activity during consecutive stages of mental rotation of 3D objects. Given the fact that the process of mental rotation of objects is associated with desynchronisation of beta rhythm (beta ERD), it was expected that suppression in this band would be greater in the experimental group than in the controls. Thirty-three participants were randomly allocated to two groups performing the classic Shepard–Metzler mental rotation task (1971). The experimental group received auditory stimuli when the level of concentration fell below the threshold value determined separately for each participant based on the engagement index [β/(α + Θ)]. The level of concentration in the control group was not stimulated. Compared to the controls, the experimental group was found with greater beta-band suppression recorded above the left parietal cortex during the early stage and above the right parietal cortex during the late stage of mental rotation task. At the late stage of mental rotation, only the experimental group was found with differences in beta ERD related to varied degrees of the rotation angle and the control condition (zero angles, no rotation) recorded above the right parietal cortex and the central area of cerebral cortex. The present findings suggest that acoustic feedback might improve the process of mental rotation.

## Introduction

Mental images are created in a process during which the experience of “inner vision” is generated in a situation when a visual stimulus is not accessible to sensory receptors (Kosslyn 2005). According to Kosslyn’s model, there are six modules of imagery formation. Each module corresponds to successive forms of imagery starting from a draft of the image through more and more detailed forms endowed with morphological and contextual features and ending with an analogue form of an object. The author of the model attributed the key role to the module of attention shifting, which enables sequential visualisation of the specific parts of an object with appropriate relations between them. Baddeley (2002) proposed that spatial imagery is based on the operation of the visuospatial sketchpad, which (like other modules of working memory) is controlled by the central executive system responsible for focusing, shifting, and allocating attentional resources to specific tasks (Hyun and Luck 2007). Analysis of the two theoretical models shows the importance of maintaining high levels of attention during the process of imagery generation.

The process of maintaining optimal attention levels is based on self-regulation involving control of one’s own mental processes (Rueda et al. 2011). One method of monitoring mental states is based on measurement of the related changes in the bioelectrical activity of the brain, for example with electroencephalography (EEG). The signal can then be consciously regulated in the process of neuronal feedback (neurofeedback, NF). The effectiveness of neurofeedback as a method enabling self-regulation of selected parameters of brain activity using visual or auditory feedback has been confirmed by several studies (Hinterberger et al. 2004; Gargiulo et al. 2012; McCreadie et al. 2014). Maintenance of high concentration on a task is enabled by combining focused attention, reflected for example by the proportion of theta and beta waves, with learning based on feedback (Carmody et al. 2001; Gruzelier 2014; Liu et al. 2018; Strehl 2014; Thompson and Thompson 2012). Increased beta-band activity is reflected by greater vigilance and cognitive involvement (Offenloch and Zahner 1990; Lubar 1991). The significant role of beta and theta bands was also demonstrated by studies focusing on participants with attention deficit disorders (Gola et al. 2013; Kropotov et al. 2005). As for the need to maintain focused attention on a task, correlations were observed between the dynamics of alpha power and errors presented in sustained attention tasks (Kelly et al. 2003; Huang et al. 2007). Furthermore, some studies report that alpha power decreases with greater cognitive effort determined by the amount of information processed (Klimesch 1997; Ray 1990; Veigel and Sterman 1993). Review of research into practical applications of cognitive neuroscience and advanced neurotechnology shows that the level of focus can be controlled by monitoring the engagement index, which is calculated based on the ratio of beta power (13–22 Hz) to the sum of alpha (8–13 Hz) and theta rhythms (4–8 Hz) [β/(α + Θ)] (Mikulka et al. 2002; Pope et al. 1995; Prinzel et al. 2000). The engagement index reflects the degree of cognitive involvement in task performance. Berka et al. (2007) demonstrated its relationship with tasks requiring attentional vigilance. Research reports also show that the index is additionally linked with the processes involving information gathering, visual scanning, and sustained attention (Berka et al. 2007; Chaouachi et al. 2010; Nuamah and Seong 2017; Hamadicharef et al. 2009).

The present study applied neurofeedback based on monitoring of the engagement index during the performance of imagery task. In our research, we used a novel approach opposite to classic neurofeedback techniques, as the warning is given when the participant’s attention is low instead of giving rewards for keeping attention above the defined threshold. Feedback provided with a sound (440 Hz, 73 dB SPL, 600 ms) was generated when the engagement index was below the threshold defined specifically for a given person for a duration of more than 3000 ms. Results of experiments investigating brain activity evoked by auditory stimuli in a few varied conditions of attention engagement show that cognitive processing is applied to sounds with a duration of 600 ms (Grimm et al. 2004, 2006). Our experiment applied sound parameters (frequency and intensity) used in the procedure proposed by Grimm et al. (2006). The imagery task was designed in accordance with the experimental procedure used by Shepard and Metzler (1971) in which participants compare two rotated three-dimensional figures and determine whether they are the same or they represent mirror images. Replication studies using the same procedure confirmed the linear relationship between the angle of rotation of the presented object and duration of mental rotation (Cooper and Shepard 1975; Jolicœur et al. 1985). The function shows a growing tendency for angles ranging from 0° to 180°. The present study was designed to use the procedure described by Schendan and Lucia (2009) in which nine angles of figure rotation were divided into three levels: low (20°, 40°, 60°), medium (80°, 100°, 120°), and high (140°, 160°, 180°). The control condition was defined as no rotation in mirror image figures or 0° rotation in the same objects. According to Heil (2002), a performance of mental rotation tasks involves several stages: perceptual encoding, identification-discrimination, identification of the object’s orientation, mental rotation, judgment of the parity, response selection, and response execution. The stages represent consecutive phases of information processing during the performance of mental rotation tasks (Chen et al. 2014; Cooper and Shepard 1975). Taking this into account, the analysis of electrophysiological data was conducted separately for the early stage (600–1000 ms), middle stage (1000–2000 ms), and late stage (2000–2500; 2400–3000 ms). The time window for the first stage was determined based on research reports suggesting that identification of the object’s orientation and mental rotation begin at approximately 450–600 ms after the exposition of Shepard–Metzler’s figures (Peronnet and Farah 1989; Riečanský and Jagla 2008; Schendan and Lucia 2009). The middle stage corresponds to the process of comparing a pair of objects, and the final stage involves selection of the correct answer and preparation for a motor response (Chen et al. 2014; Cooper and Shepard 1975).

Findings of fMRI studies suggest that the parietal lobes are mainly involved in generation and manipulation of mental representations (Podzebenko et al. 2002; Schendan and Stern 2007; Zacks 2008). Other studies also found activation in motor areas (Bode et al. 2007; Lamm et al. 2007; Richter et al. 2000). In EEG studies, such locations cannot be directly identified, but changes in the intensity of beta-band signal are recorded from the leads placed above these areas of the cerebral cortex. Time–frequency characterisation of brain activity during performance of the Shepard–Metzler task shows a dominant role of beta-band suppression, although this effect was usually linked to tasks engaging motor imagery (Engel and Fries 2010; Nam et al. 2011; Wang et al. 2016). Simultaneously conducted fMRI and EEG measurements showed a significant relation between beta ERD and the process of mental transformation of objects (Sasaoka et al. 2014). Sasaoka and colleagues established that increased activation in bilateral parietal cortex and the left premotor cortex is accompanied with greater beta-band suppression during the mental transformation of objects. Beta-band desynchronisation increases with the amount of mental transformation applied to an object; the relevant evidence was reported for the number of transformations (Sasaoka et al. 2014) and for the angle of rotation (Chen et al. 2014).

Taking into account the role attributed by contemporary theories (Baddeley 2002) to attention involved in the process of generation and manipulation of mental representations, it was assumed that increased focus would foster imagery generation during the performance of object-based mental rotation tasks. The purpose of the present study was to identify the effect of acoustic neurofeedback on brain activity during consecutive phases of mental rotation of 3D objects. Given the fact that the process of rotating imagery objects is associated with beta ERD in the central and parietal areas, it was expected that suppression in this band would be greater in the experimental group than in the controls. The analyses also examined the effect of interaction between the variables of group and rotation angle.

## Method

### Participants

At the initial stage of the project, the study group consisted of 39 individuals. All participants were volunteers and agreed to participate in the study; they were informed about the possibility to resign from participation at any time without stating the reasons. The experiment was conducted in compliance with the Declaration of Helsinki. The participants were randomly selected to the study group. The initial selection of the participants was performed based on questions of a specially designed survey related to neurological injuries, attention deficits, and the performance of job-related tasks engaging spatial imagery (improving the related capacities). Because the experimental procedure applied auditory stimuli, the participants were participated to audiometric tests. Pure tone audiometry testing based on the Hughson-Westlake method for threshold assessment was performed using an AD629 diagnostic audiometer (Interacoustics, Denmark). Participants with diagnosed attention deficits and with hearing impairments as well as those performing jobs engaging spatial abilities were excluded from the study. Ultimately, following the final selection stage related to the quality of the recorded EEG data, 33 participants (17 females) were qualified for the study. Their mean age was approximately 32 years (*M* = 32.06; *SD* = 7.67). Individual factors potentially affecting the results were measured to examine the homogeneity of the experimental group and the control group. The applied tools included two tests from the Vienna Test System, the DAUF (Sustained Attention Tests) and the A3DW (Adaptive Spatial Ability Test), as well as the AMI (Achievement Motivation Inventory; Klinkosz and Sękowski 2013). The participants’ chronotype was assessed using survey questions based on selected items from the Morningness–Eveningness Questionnaire (MEQ) to determine their compatibility with the timing of the experiment (Hone and Ostberg 1976). The characteristics of the controlled variables are presented in the Appendix Tables 1 and 2.

### Equipment and Software

Measurement of bioelectrical brain activity during the experiment was performed using a Mitsar 202 system (Mitsar Co. Ltd., Saint Petersburg, Russia) with a recording range from 0.16 to 70 Hz. The signal was recorded using a 19-channel cap with passive Ag/AgCl electrodes. The study applied references for the left ear electrode A1 for all channels. Examinations were conducted at impedance maintained below 5 kΩ and sampling frequency of 500 Hz. Visual inspection of EEG signal and monitoring of impedance were performed using EEG Studio Acquisition ver. 1.10.10. The data were forwarded via LSL (Lab Streaming Layer) library to Mitsar Recorder software, which computed the engagement index [β/(α + Θ)] in real time and generated a sound if the index fell below a defined threshold value. The software designed for presentation of experimental stimuli was written in C++ language and was synchronised using a photosensitive diode connected directly to the EEG amplifier.

During the experiment, the stimuli were displayed on a BENQ BL902 TM monitor with a diagonal of 19 inches, resolution of 1280 × 1024 pixels, and response time of 5 ms. The participants’ distance from the screen was 86 cm. Behavioural indexes were controlled with a Logitech keyboard (model Y_UY95). Instrumental biases resulting from the transfer of information between the specific equipment components and software were controlled.

### Procedure

The trials were conducted individually in the Experimental Psychology Laboratory at the University of Economics and Innovation in Lublin. During the mental rotation of objects, the participants from the experimental group could hear a sound signal when the level of their concentration decreased. A tone with a frequency of 440 Hz, an intensity of 73 dB SPL, and duration of 600 ms was generated when the engagement index was below the threshold individually determined for a given person for a duration of more than 3000 ms. The sound was emitted in a free field from loudspeakers with a high frequency range (PS-HC2-1, Samsung Electronics Co., Ltd., Suwon, South Korea). The participants from the experimental group were instructed at the start that after they heard this sound they should try to focus their attention again. In the control group, no neurofeedback was used during the mental rotation 3D objects. The differences in the test situation between the experimental and control groups (sound emission versus lack of sound, different instructions) resulted in the impossibility of applying the double-blind method. The researcher could not influence the results because they were not present during the registration in the same place as the participant. In addition, the participants’ data were coded at the analysis stage.

**Fig. 1:**
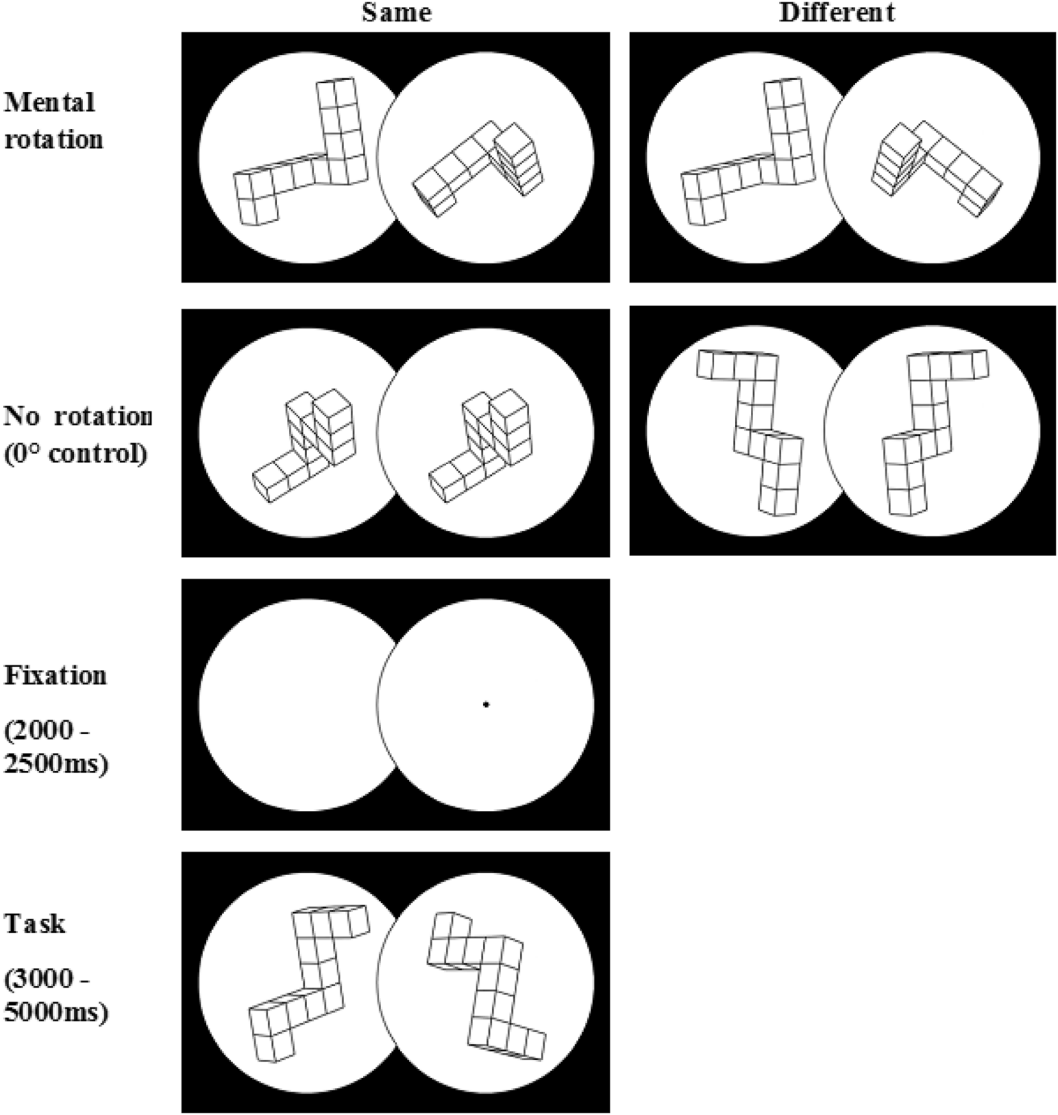
Experimental procedure and stimulus material

The stimuli consisted of two objects built from cubes placed inside two circles with a white background. The circles were partly overlapping, and the rotated object was placed on the right side at the central point of eye fixation. The optimal size of the stimulus was defined as a visual angle of 9° horizontally, 5° vertically, and a distance of ~ 86 cm from the monitor. The present study was designed to use the procedure described by Schendan and Lucia (2009) in which nine angles of figure rotation were divided into three levels: low (20°, 40°, 60°), medium (80°, 100°, 120°), and high (140°, 160°, 180°). The control condition was defined as no rotation in mirror image figures or 0° rotation in the same objects. A fixation panel was displayed following exposition of each stimulus, which consisted of two circles without any figures. The central fixation point, presented as a dot, was located in the middle of the circle on the right (Fig. 1).

### Training

The training procedure directly preceded the experiment and was based on stimuli analogous to those used during the trials. The training differed from the experimental trials in the number of presented stimuli (18) and the angle of their rotation (from 10° to 170°). Additionally, the participants received feedback related to the correctness of responses. This information (correct/incorrect) was displayed after the stimulus exposure. Based on the engagement index values recorded during the training sessions, a mean was computed separately for each participant from the experimental group. During the trial, the mean was applied as the threshold value.

### Experiment

After the training session, each participant received the instructions again. The duration of its presentation was not limited. The participants were asked to press a specified key when the two objects on display were the same and another key if the object on the right was a mirror image of the figure presented on the left, regardless of the angular difference. The experiment comprised 144 trials, 72 in the control condition with no rotation and 72 in the condition with rotation. All trials were displayed in one experimental block (a mixed design was applied in accordance with the procedure described by Schendan and Lucia 2009). Rotation trials and control trials were displayed in a pseudo-randomized way in the order applied in the experimental procedure described by Amick et al. (2006). No more than three trials requiring the same response could be displayed in sequence. This principle was applied alike to three consecutive responses of “the same objects” and three consecutive responses of “mirror images.” Another rule was that an object with the same angle of rotation could not be displayed again until all objects with the remaining angles of rotation had been presented (Kosslyn et al. 1998). Fixation panels preceding the display of pairs of stimuli were shown for a duration of 2000–2500 ms in a random manner, and a pair of figures was displayed for 3000 ms or until a response was given (but no longer than 5000 ms). After the stimulus was on display for 3000 ms, a dot appeared in the middle of the area shared by the two circles, and this informed participants that they could provide a response. This procedure was applied to prevent muscle related artefacts in the data analysed in the selected time windows.

### Data Analysis

After the end of the trial, electrophysiological data were analysed using the package EEGLab v13.5.4b, which is an extension for MATLAB R2015a (*MathWorks*, Natick, MA, USA). High-pass filters over 0.5 Hz and low-pass filters below 40 Hz were applied. Cleanline was used to correct the recording by the frequency of 50 Hz corresponding to the power line operation. Subsequently, independent component analysis (ICA) was performed, and then ocular artefacts (*EOG*) and muscular artefacts (*EMG*) present among the independent components were removed manually (Jung et al. 2000a, b). The data prepared in this way were divided into 3000 ms segments (from 0 to 3000 ms), and the baseline was calculated from 0 to − 1000 ms. To calculate the strength of the signal (dB) for the entire window (three-cycles; 0.5 s) the segments were subjected to time–frequency decomposition with ERSP (Event-Related Spectral Perturbation; Makeig 1993).

The mean results calculated for the experimental conditions were exported to *SPSS Statistics 21* (IBM, Inc., USA). Comparative analyses examining the effects of acoustic feedback during mental rotation took into account the electrodes covering the parietal cortex (P3, Pz, P4) and central areas (C3, Cz, C4). Frequency ranges were selected based on visual inspection of the differences between conditions indicated by time–frequency diagrams. The analyses took into account frequency ranges in the beta-band (18–20 Hz; 20–24 Hz). Since the purpose of the study was to investigate the effects of acoustic neurofeedback at the consecutive stages of mental rotation, four time windows were selected for the calculations in order to present changes occurring in time (600–1000 ms; 1000–2000 ms; 2000–2500 ms; 2400–3000 ms). The data were analysed in relation to the three levels of figure rotation: low (20°, 40°, 60°), medium (80°, 100°, 120°), and high (140°, 160°, 180°), also taking into account the control condition (0°) (Schendan and Lucia 2009).

## Results

In order to examine behavioural data, a two-way mixed-design analysis of variance (4 × 2) was conducted with the intra-object factor Rotation Angle (low, medium, high, 0° control condition) and the inter-object factor Group (experimental, control). Analysis of data representing accuracy of the responses did not show a statistically significant main effect of Group, *F*(1, 31) = 0.48, *p* = 0.493, *η*^2^ = 0.01, nor an interaction effect of Group and Rotation Angle, *F*(1, 31) = 1.99, *p* = 0.120, *η*^2^ = 0.06.

Two-way mixed-design analysis of variance (4 × 2) with the intra-object factor Rotation Angle and the inter-object factor Group was conducted separately for two ranges of the beta-band, 18–20 Hz and 20–24 Hz. Post-hoc comparisons were conducted using Bonferroni correction for multiple comparisons.

### First Stage (600–1000 ms)

**Fig. 2:**
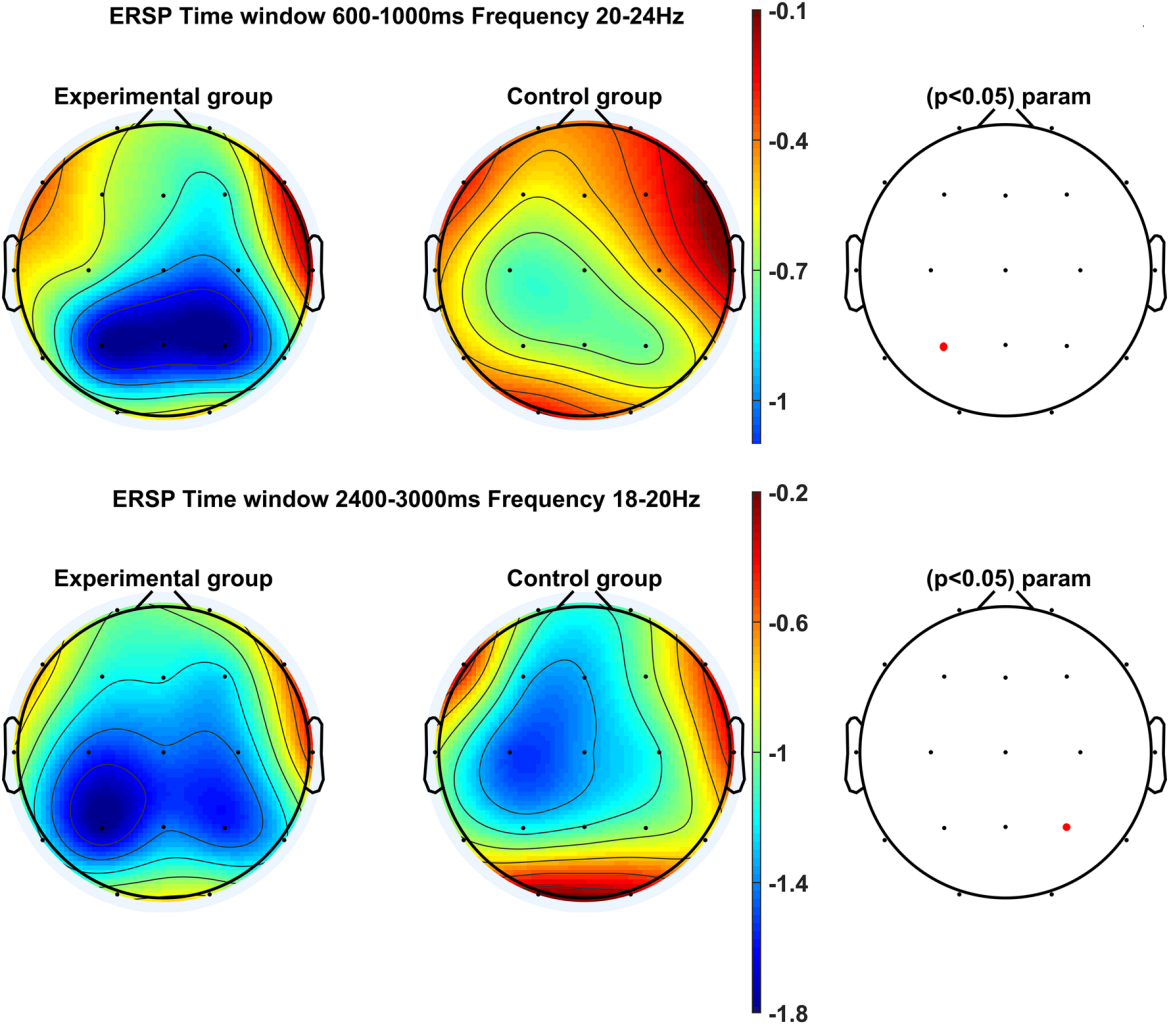
Maps showing the distribution of signal strength on the skull at the early (top, 600–2400 ms) and the late (bottom, 2400–3000 ms) stage during the performance of mental 3D object rotation task. Desynchronisation in beta-band (18–20 Hz; 20–24 Hz) is marked in blue. Significant differences in signal strength between the experimental (left) and the control group (right) are shown by the electrons marked in red. (Color figure online)

**Fig. 3:**
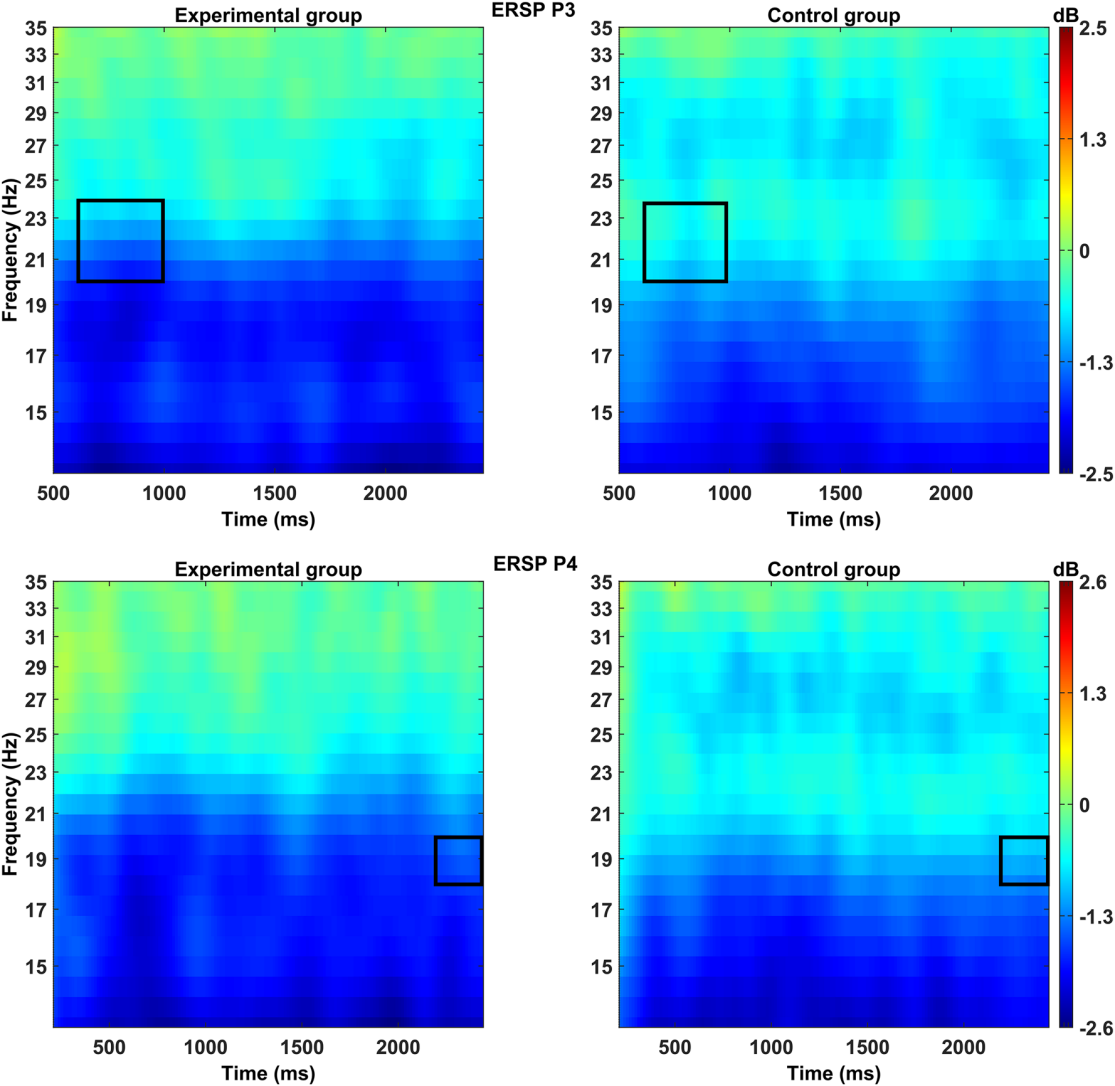
Time–frequency diagram showing ERD recorded during mental rotation of 3D objects over the left (P3) and the right (P4) parietal lobe. The rectangular contour delineates the frequency–time range in which significant differences occurred between the experimental group and the controls

Time–frequency analysis conducted for the early stage of the mental rotation task (600–1000 ms) showed a significant main effect of Group in the left parietal cortex (P3), *F*(1, 31) = 4.13, *p* = 0.05, *η*^2^ = 0.12. The experimental group (*M* = − 1.27 dB, *SD* = 1.08) achieved significantly stronger ERD in the frequency range of 20–24 Hz compared to the controls (*M* = − 0.61 dB, *SE* = 0.79; Figs. 2, 3).

### Second Stage (1000–2000 ms)

No significant main effects or interactions related to the groups were found for the middle stage of the mental rotation task (1000–2000 ms).

### Third Stage (2000–3000 ms)

In the time window of 2000–2500 ms, a significant interaction effect was found for Group × Rotation Angle over the right central area of cerebral cortex (C4), *F*(1, 31) = 3.44, *p* = 0.020, *η*^2^ = 0.10. Multiple post-hoc comparisons with Bonferroni correction showed that in the experimental group, the strength of desynchronisation at the frequency range of 18–20 Hz was significantly greater for a medium rotation angle (*M* = − 2.12 dB, *SD* = 1.33) compared to the 0° control condition (*M* = − 1.17 dB, *SD* = 1.20).

**Fig. 4:**
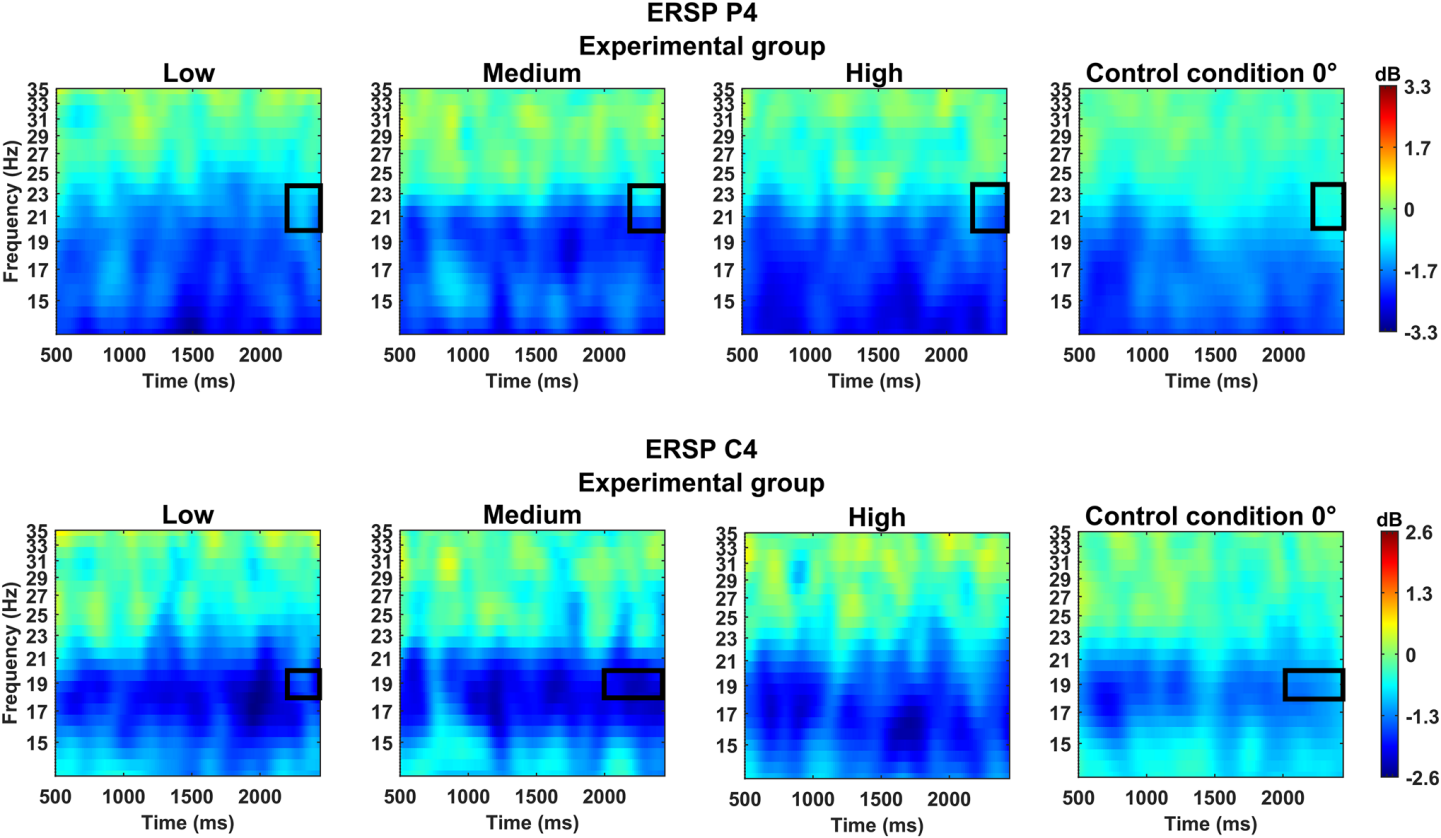
Time–frequency diagrams showing ERD recorded over the right parietal lobe (P4, top), and over the right central cerebral cortex (C4, bottom), during mental rotation of 3D objects. The rectangular contour delineates the frequency-time range in which significant differences occurred between the level of mental rotation of the figures (low, medium, high, 0°) in the experimental group

**Diagram 1:**
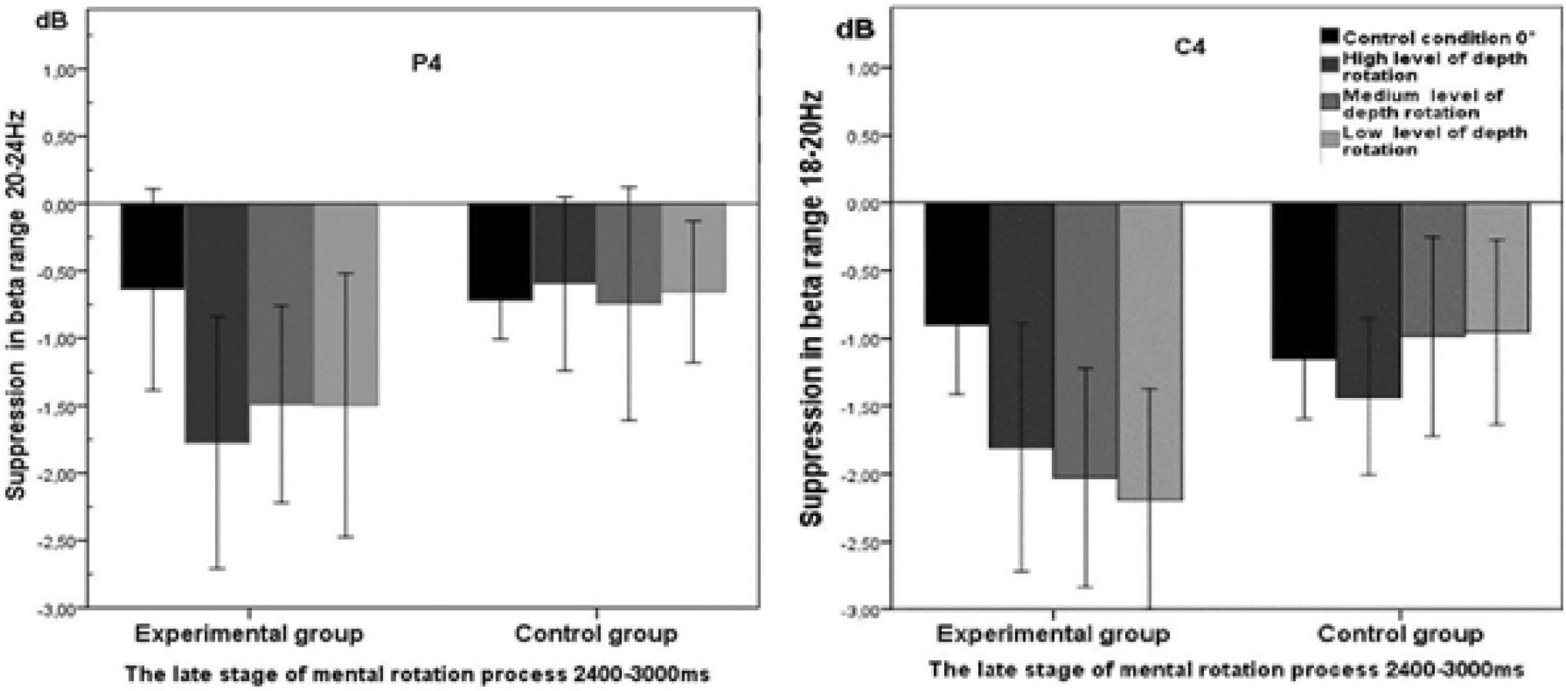
Differences in beta-band desynchronisation related to the effect of Group × Rotation Angle during the late stage of mental object rotation (2400–3000 ms). The differences in the strength of the signal recorded from the lead C4 (18–20 Hz) and from the electrode P4 (20–24 Hz) are shown on the left and on the right, respectively. The vertical columns represent the standard error of the mean

During the late stage of the mental rotation task (2400–3000 ms), there was a significant main effect of Group in the right parietal cortex (P4), *F*(1, 31) = 5.09, *p* = 0.031, *η*^2^ = 0.14. The experimental group (*M* = − 1.86 dB, *SD* = 1.29) presented a significantly greater decrease in the strength of waves at the frequency range of 18–20 Hz compared to the control group (*M* = − 0.97 dB, *SD* = 0.97; Figs. 2, 3). In the same time window, there was also a significant interaction effect of Group × Rotation Angle in the right parietal cortex (P4), *F*(1, 31) = 3.34, *p* = 0.023, *η*^2^ = 0.10. Post-hoc analysis showed differences in the strength of suppression in the frequency range of 20–24 Hz at the high rotation angle (*M* = − 1.77 dB, *SD* = 1.76), medium rotation angle (*M* = − 1.63 dB, *SD* = 1.32), low rotation angle (*M* = − 1.50 dB, *SD* = 1.83), and the control condition (*M* = − 0.64 dB, *SD* = 1.40) only in the experimental group (Fig. 4; Diagram 1).

In the time window of 2400–3000 Hz, there was a significant interaction effect for Group × Rotation Angle based on the central leads (C4), *F*(1, 31) = 3.10, *p* = 0.031, *η*^2^ = 0.09. Post-hoc analysis showed that in the experimental group, the strength of desynchronisation in the frequency range of 18–20 Hz was significantly greater for the low (*M* = − 2.19 dB, *SD* = 1.65) and medium angles of rotation (*M* = − 2.03 dB, *SD* = 1.62) compared to the 0° control condition (*M* = − 0.90 dB, *SD* = 1.03; Fig. 4; Diagram 1).

## Discussion

The present study was designed to determine the effect of acoustic neurofeedback on brain activity during consecutive stages of a 3D-object-based mental rotation task. As expected, the strength of beta-band desynchronisation in the parietal cortex was greater in the experimental group than in the control group. In the early stage of the mental rotation task, the group receiving acoustic neurofeedback presented significantly stronger beta-band (20–24 Hz) desynchronisation above the left parietal cortex compared to the controls. On the other hand, during the late stage of the task, the experimental group presented stronger beta-band (18–20 Hz) suppression above the right parietal cortex than the control group. The differences in beta ERD related to interactions with the degree of rotation angle were only observed in the experimental group at the late stage of mental rotation. Above the right parietal cortex, there was a significantly greater decrease in wave strength in the beta band (20–24 Hz) for the high, medium, and low degrees of the rotation angle compared to the control condition. On the other hand, the strength of beta-band (18–20 Hz) desynchronisation above the right central area of the cerebral cortex was significantly greater for the medium rotation angle than for the control condition.

In a discussion focusing on the significance of the present findings, one cannot disregard the concept of the engagement index, which is mainly used in neuro-ergonomics research (Freeman et al. 1999; Mikulka et al. 2002; Offenloch and Zahner 1990). To our knowledge, no studies conducted so far have utilised the engagement index to examine the impact of acoustic neurofeedback on mental rotation of 3D objects. Given the above, the relationships observed in the present findings are explained by reference to studies presenting the dynamics of cortical activity during mental image transformation.

The results related to increased beta suppression enabled by neurofeedback above the left parietal cortex at the early stage and above the right parietal cortex at the late stage of mental rotation are consistent with the findings reported by Sasaoka et al. (2014). Using simultaneous EEG and fMRI measurements, these researchers established that the process of mental image transformation is accompanied by beta-band desynchronisation observed bilaterally in the parietal cortices. Based on those research findings, the effect of increased beta-band suppression in the experimental group that we observed might suggest that neurofeedback reinforces the process of mental rotation of objects. The fact that the effects were observed above the right and left parietal cortex might result from the specificity of the procedure that involved comparison of the object before and after the rotation to the figure on the right side. This might also be linked with the high level of task difficulty. Sack et al. (2005) showed that the left parietal cortex is predominant in generating mental images, while the right parietal cortex is involved in the process of spatial comparison. Mental rotation of figures, as described by Shepard and Metzler, activates parietal regions to a degree on both sides, and bilateral involvement of the structures might increase with growing requirements of a rotation task or depending on the procedure used in the study (Cohen et al. 1996; Corballis 1997; Kosslyn et al. 1998; Tagaris et al. 1997; Zacks 2008). The differences between the groups at the early stage of the mental rotation task (600–1000 ms) are related to the phase involving identification of the object’s orientation and its mental rotation (Peronnet and Farah 1989; Riečanský and Jagla 2008; Schendan and Lucia 2009). At the late stage (after rotation), the object is compared to the figure on the right side, and the response is selected (Heil 2002). It has been shown that an increase in rotation angle or number of transformations coincides with greater suppression of beta-band power (Chen et al. 2014; Engel and Fries 2010). The decrease in beta-band power depends on the number of mental transformations and is accompanied with the bilateral activity of the parietal cortex and the left premotor cortex (Sasaoka et al. 2014). These reports are in line with the interaction effects identified by our study involving the right parietal cortex and the central area of the cerebral cortex. Differences in beta ERD related to the varied degrees of the rotation angle and the control condition were observed during the late stage of mental rotation task only in the experimental group. In the context of the above studies reporting relationships between beta-band suppression and rotation angle, the effect we identified can be interpreted to suggest that neurofeedback reinforces the process of object comparison and response selection only if the objects were previously mentally rotated. The central area, for which one of the effects was found in the neurofeedback group, corresponds functionally to the motor and premotor cortex, which plays an important role in the parieto-premotor network activated during mental object transformation (Sasaoka et al. 2014; Seurinck et al. 2011). Sasaoka et al. (2014) suggest that the signal from the premotor cortex is used in mental image transformation in the parietal areas and in updating the mental representation in the right posterior parietal cortex. According to an explanation offered by these researchers, beta-band desynchronisation increasing in the premotor cortex with the rotation angle might be linked with motor strategies of mental rotation utilised by the participants. Some studies available in the literature present evidence that the motor cortical areas are involved in the process of mental rotation regardless of the strategy applied (Bode et al. 2007). However, according to most research reports, these regions are activated only during the process of using the internal strategy of imagining to rotate objects with one’s own hands (Horst et al. 2012, 2013; Kosslyn et al. 2001). Nam et al. (2011) demonstrated that beta-band desynchronisation is closely linked to motor processes and imagery of movement. In this context, surprising findings were reported by Wang et al. (2016), who recorded stronger beta-band desynchronisation above the left central and right fronto-central areas in a group utilising an external strategy compared to an internal strategy. On the other hand, analyses conducted by Chen et al. (2014), who compared beta ERD in groups using internal and external mental strategies, did not identify significant differences between the groups. Given the disparities in the related evidence, this aspect should be investigated in controlled studies in the future. Attempts to explain the late effect observed in the motor areas should take into account the evidence suggesting that beta-band desynchronisation is associated with real or imagined movement and in particular with motor planning (Alegre et al. 2003; Kaiser et al. 2001; Kilner et al. 2005; Klostermann et al. 2007; Tzagarakis et al. 2010; Zapała et al. 2018). At the final stage before responding, the participant compares the objects and chooses the answer, preparing to press the appropriate key. Taking this into account, it is likely that the late effect above the motor areas reflects the process of movement planning. In other words, the change in the signal intensity in this frequency range might suggest that neurofeedback strengthens the preparation for responding, in particular in the case of the moderate difficulty level of the rotation task.

Significant limitations of the present study result from the small sample size and the fact that the measurements were performed during single sessions. In this context, it was difficult to expect that neurofeedback would affect the accuracy of performance in the mental rotation task. The study procedure with the defined duration of exposition to the mental rotation task, after which the response was to be given, did not allow us to examine the temporal aspect of responding but only the accuracy of responses. Because the EEG measurement was performed using a cap with a small number of channels, application of methods for locating the source of signal would have posed a risk of significant error. The identified limitations of the experiment suggest that further research should involve a larger sample and should apply high-density EEG. Another limitation is the lack of a sham control group in which the same sound would be generated in a way that is not related to the level of the engagement index. In subsequent studies, it is worth considering three groups: an experimental group with neurofeedback, a sham control group with a randomly generated sound, and a control group without emitted sound. Furthermore, a future study could be designed to verify the obtained effects during a series of neurofeedback sessions. This approach would make it possible to check whether strengthening of beta ERD in the parietal areas during a single neurofeedback session translates into improved accuracy after training. Furthermore, by designing another experiment with a modified procedure without predefined duration of the stimuli exposition, it would be possible to identify the impact of such training on the time-related effectiveness of mental rotation of 3D objects. Additionally, it would be interesting to see whether the effect of strengthened beta ERD associated with movement planning in the central areas would also be observed if the modified procedure was applied.

In summary, the present study showed that stronger beta-band suppression was recorded in the experimental group compared to the control group above the left parietal cortex at the early stage and above the right parietal cortex at the late stage of a mental rotation task. Only the experimental group was found with differences in beta ERD related to the varied degrees of the rotation angle and the control condition identified above the right parietal cortex and the central area of cerebral cortex. The present findings suggest that acoustic neurofeedback might strengthen beta ERD at the early and late stages of mental rotation and consequently improve the process. It is well established that visuospatial abilities, in particular skills of mental object rotation, are of key importance for pilots or car drivers (Dror et al. 1993; Kosmidis et al. 2014). Our discovery opens the door for the verification of the effects obtained in our study during a series of neurofeedback sessions for people using spatial imagination in their professional work.

## Appendix

See Tables 1 and 2.

**Table 1:** Achievement motivation, spatial imagination and sustained attention characteristics for experimental and control group

Controlled variables	Experimental group		Control group		*t*	*p*
	*M*	*SD*	*M*	*SD*		
Total score AMI	783.81	67.34	794.82	97.60	− 0.38	0.710
FX—flexibility	49.50	7.60	49.94	6.95	− 0.17	0.863
FE—fearlessness	36.00	5.49	38.88	6.53	− 1.37	0.181
PD—preference for difficult tasks	46.88	7.96	47.94	8.06	− 0.38	0.705
ID—independence	49.25	7.83	49.53	7.86	− 0.10	0.919
CS—confidence in success	47.88	8.67	51.00	9.39	− 0.99	0.290
DO—dominance	41.50	4.70	42.18	9.27	− 0.27	0.792
EA—eagerness to learn	46.56	6.15	46.29	7.03	0.12	0.908
GS—goal setting	49.44	7.92	50.12	10.52	− 0.21	0.836
CE—compensatory effort	47.63	6.70	45.47	12.95	0.61	0.551
SO—status orientation	48.00	5.01	44.65	8.54	1.39	0.177
EN—engagement	44.56	6.43	41.24	10.44	1.11	0.277
PP—pride in productivity	53.75	6.73	56.76	8.62	− 1.12	0.273
CO—competitiveness	41.38	6.02	40.71	8.99	0.25	0.802
FL—flow	49.75	7.84	53.82	11.15	− 1.21	0.237
IT—internality	46.75	3.77	47.71	5.67	− 0.57	0.575
PE—persistence	42.44	5.50	41.94	4.35	0.29	0.775
SC—self-control	43.75	4.91	47.64	7.19	− 1.81	0.081
DAUF response time	0.70	0.11	0.80	0.14	− 2.18	0.037
DAUF accuracy	118.7	1.35	117.88	2.47	1.15	0.259
A3DW spatial imagination quotient	81.25	18.10	85.18	15.88	− 0.65	0.523

**Table 2:** Characteristic of compliance of chronotype with the time of day of research for experimental and control group

Compliance of chronotype with the time of day of research	Experimental group		Control group		% of the cumulative value	χ^2^	*p*
	*N*	%	*N*	%			
Congruent	8	24.2	9	27.3	51.5	0.29	0.866
Incongruent	8	24.2	8	24.3	48.5		
